# Role of STAT1 in modulating the host immune response to *Plasmodium yoelii* 17XL-infected murine blood-stage malaria

**DOI:** 10.1128/spectrum.03032-25

**Published:** 2026-03-30

**Authors:** Yifan Sun, Chenyan Du, Qinwen Xu, Zhe Chen, Xin Feng, Su Han, Yan Yan, Bo Wang, Zhiyue Lv, Guoding Zhu, Jianping Cao, Yang Cheng

**Affiliations:** 1 Laboratory of Pathogen Infection and Immunity, Department of Public Health and Preventive Medicine, Jiangnan University, Wuxi School of Medicine 66374, , Wuxi, Jiangsu, China; 2 Department of Laboratory Medicine, Affiliated Hospital of Jiangnan University 199193, Wuxi, Jiangsu, China; 3 School of Food Science and Technology, Jiangnan University 66374, Wuxi, Jiangsu, China; 4 Laboratory for Infection and Immunity, Affiliated Hospital of Jiangnan University, The Fifth People’s Hospital of Wuxi 199193, Wuxi, Jiangsu, China; 5 Hepatology Institute of Wuxi, The Fifth People’s Hospital of Wuxi, Affiliated Hospital of Jiangnan University, Wuxi, Jiangsu, China; 6 Department of Clinical Laboratory, The First Affiliated Hospital of Anhui Medical University 36639, Hefei, Anhui, China; 7 Zhongshan School of Medicine, Sun Yat-sen University, Guangzhou, Guangdong, China; 8 Key Laboratory of National Health and Family Planning Commission on Parasitic Disease Control and Prevention, Jiangsu Provincial Key Laboratory on Parasite and Vector Control Technology, Jiangsu Institute of Parasite Diseases, Wuxi, Jiangsu, China; 9 National Institute of Parasitic Diseases, Chinese Center for Disease Control and Prevention (Chinese Center for Tropical Diseases Research), NHC Key Laboratory of Parasite and Vector Biology, WHO Collaborating Centre for Tropical Diseases, National Center for International Research on Tropical Diseases, Shanghai, China; 10 Institute for Prevention and Control of Tropical Diseases and Chronic Diseases, Hainan Provincial Center for Disease Control and Prevention (Hainan Academy of Preventive Medicine), Haikou, Hainan, China; University of Edinburgh, Midlothian, United Kingdom

**Keywords:** Malaria, STAT1, IFN-γ, CD8^+^T cell, anemia, *Plasmodium yoelii*

## Abstract

The spleen is critically involved in the immune response to malaria parasite infections. However, the precise molecular mechanisms underlying splenic immune regulation and anemia during malaria remain elusive. This study aimed to identify the key regulatory proteins in *Plasmodium yoelii* (Py)-infected host spleen and investigate its role in malaria pathogenesis. Notably, significantly upregulated proteins in *Py*17XL-infected mice spleens were enriched in interferon (IFN) signaling, with signal transducer and activator of transcription (STAT)1, a critical component of the IFN pathway, emerging as the core molecule in the interaction network. Subsequent validation confirmed marked STAT1 upregulation in the spleens of infected hosts. Genetic ablation of STAT1 enhanced mouse survival rates and substantially reduced peripheral parasitemia post-infection and splenic parasite burden. Furthermore, STAT1 deficiency markedly reduced proportions of IFN-γ-producing CD8^+^ T cells and interleukin-10-producing CD4^+^ T cells at day 7 post-infection. Interestingly, hemoglobin concentrations and the proportion of peripheral blood reticulocytes significantly increased in STAT1-knockout mice compared with those in wild-type mice, indicating that STAT1 deficiency may alleviate malarial anemia. Altogether, the findings of this study elucidate the impact of dynamic protein changes, particularly STAT1, in the host spleen during malaria and provide a research basis for developing therapeutic interventions for malaria.

**IMPORTANCE:**

The cumulative findings demonstrate the pathogenic role of STAT1 in host spleen during *Py*17XL parasite infection, highlighting its role in promoting IFN-γ production and exacerbating malarial anemia. This study offers novel insights into the key pathogenic mechanisms observed in the host spleen during malaria, providing potential therapeutic targets for malaria.

## INTRODUCTION

In 2024, there were an estimated 282 million malaria cases and 61,0000 deaths worldwide (1). These statistics highlight the importance of investigating the role of key signaling molecules in the host immune response in malaria pathogenesis for developing effective prevention and treatment strategies. The spleen is a vital immune organ and plays a critical role in clearing infected and uninfected red blood cells, along with serving as a key site for activating innate and adaptive immune responses during malaria infection (2, 3). Notably, murine models are well-established *in vivo* systems for investigating malarial immunopathology (4). Mass spectrometry (MS)-based proteomics offers an unbiased approach to profile differentially expressed proteins involved in biological functions.

The signal transducer and activator of transcription (STAT) family proteins act as downstream targets of Janus tyrosine kinases (JAKs). Furthermore, the JAK–STAT1 signaling is a crucial pathway in host immune defense against pathogen infections and regulates interferon (IFN) production and immunoglobulin class switching (5). In the classical STAT1 pathway, IFN binds to its transmembrane receptor, activating JAK1/2 and tyrosine kinase 2, which, in turn, phosphorylate STAT1, thereby leading to transcription of IFN-stimulated genes (6). Although STAT1 has been extensively studied in viral infections, its role in malaria remains elusive. STAT1 was significantly activated in *Plasmodium yoelii* (Py)17XL-infected mice spleens (7). Similarly, STAT1 was induced in the host spleen 3 days after *Plasmodium berghei* ANKA (*Pb*ANKA) infection and in both the blood and spleen 4 days after *Plasmodium chabaudi* AS (*Pc*AS) infection (8, 9). Reportedly, children susceptible to malaria exhibit upregulated STAT1 expression compared with that in protected children before the transmission season (10), underscoring the role of baseline STAT1 differences in malaria susceptibility. In malaria pathogenesis, IFN-γ, primarily produced by T cells and natural killer (NK) cells, plays a significant role in immune defense, and its overexpression drives pathological consequences (11). IFN-γ deficiency has been shown to reduce peripheral parasitemia and mitigate renal histopathological changes in *Pb* NK65-infected mice, indicating the contribution of IFN-γ to murine malaria pathogenesis (12). Conversely, interleukin (IL)-10, mainly produced by CD4^+^ T cells in malaria (13), acts as an important anti-inflammatory cytokine that counterbalances excessive inflammation (14).

Anemia is a hallmark complication of malaria, with severe malarial anemia (SMA; hemoglobin [Hb] < 5.0 g/L) as a major cause of malaria-related mortality. STAT1 is closely associated with anemia in various diseases (15–17). For instance, STAT1 expression positively correlates with anemia in patients with systemic lupus erythematosus (SLE) (16). Furthermore, patients with STAT1 gain-of-function (GOF) mutations can exhibit severe, recurrent anemia and are often diagnosed with pure red cell aplasia (15). However, the role of STAT1 in the context of erythropoiesis in malaria remains unexplored.

This study employed proteomics to screen for malaria-associated differentially expressed proteins by systematically comparing the splenic proteomes of *Py*17XL-infected mice and uninfected controls. Herein, STAT1 upregulation was observed in the infected group. STAT1 deficiency improved survival rates, reduced peripheral parasitemia, and splenic parasite burden. These results highlight the pivotal pathogenic role of STAT1 in malaria. Mechanistically, STAT1 knockout (KO) reduced IFN-γ production via splenic CD3^+^ CD8^+^ T cells and increased hemoglobin levels. Overall, this study identifies STAT1 as a critical upregulated protein in malaria-infected host spleens, elucidates its role in IFN-γ regulation and malaria anemia, and provides novel insights into the pathogenic molecular mechanisms of malaria for developing targeted intervention.

## RESULTS

### Proteomics reveals increased splenic STAT1 expression in the murine malaria model

**Fig 1:**
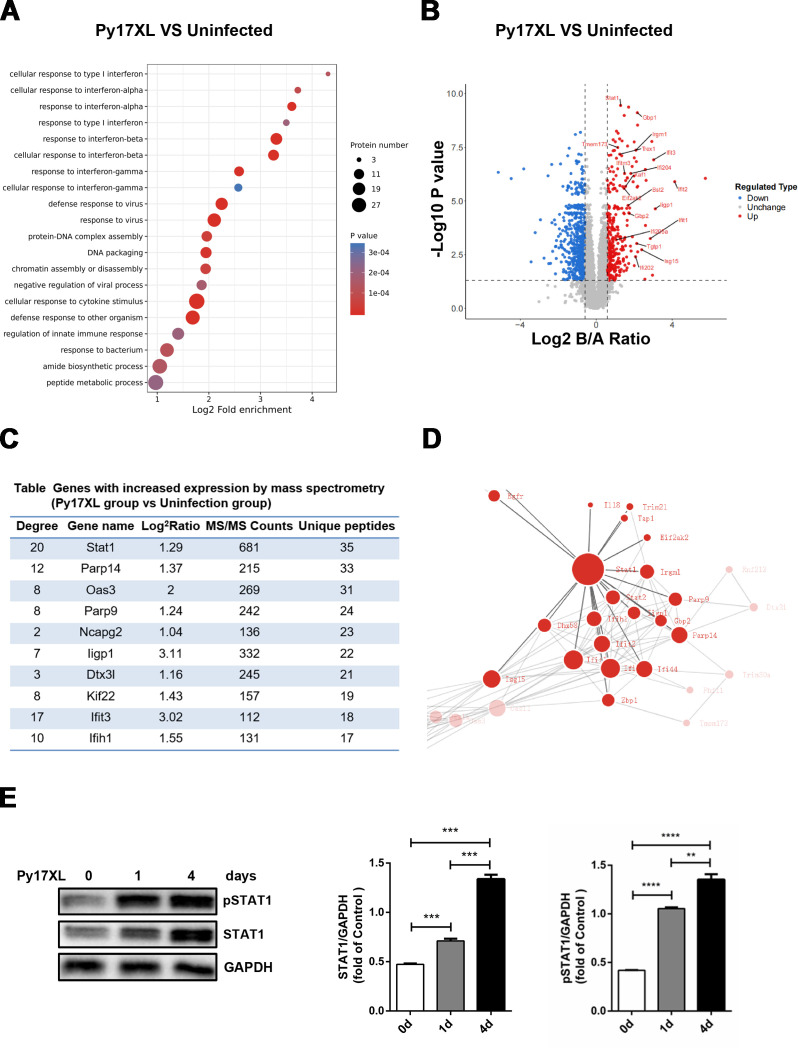
Proteomics revealed that the STAT1 expression increased significantly in the spleen of the murine malaria model. Mice (*n* = 5/group) of age 6–8 weeks were injected with 1 × 10^6^ infected RBCs (iRBCs) intraperitoneally, and their spleen tissue samples were collected on day 4 of the infection for proteomic analysis; spleen tissues from uninfected mice served as controls. (**A**) GO analysis. (**B**) Volcano plot of differentially expressed proteins between uninfected and *Py*17XL-infection groups. The significantly upregulated genes are marked in red. (**C**) List of genes showing significantly upregulated expression of proteins in the spleen of the *Py*17XL-infection group by mass spectrometry. (**D**) Upregulated protein interaction network in the spleen of infected *Py*17XL mice. (**E**) Expression of STAT1 and pSTAT1 was detected by western blotting in the spleen of *Py*17XL-infected mice on days 0, 1, and 4 post-infection. Statistical analysis of the gray values of the STAT1 expression in total protein; GAPDH served as a quantitative control for analysis. Gray values of the protein bands were quantified using ImageJ. One-way ANOVA was used for statistical comparisons. **P < 0.01; ***P < 0.001; ****P < 0.0001. Experiments were repeated 3–4 times.

To investigate changes in splenic protein expression during malaria infection, proteomics- and bioinformatics-based analyses were performed. They revealed the association of upregulated proteins with IFN responses in the *Py*17XL murine malaria model (Fig. 1A and B). Compared with uninfected controls, STAT1 showed the largest number of unique peptides in *Py*17XL-infected mice (Fig. 1C) and occupied a central position in the interaction network of upregulated proteins (Fig. 1D). The levels of STAT1 and phosphorylated STAT1 (pSTAT1) in mouse spleens were significantly higher at days 1 and 4 post-infection than at day 0 (Fig. 1E), further validating the proteomics findings. These results collectively demonstrate significant STAT1 upregulation in the host spleen during malaria infection.

### STAT1 deficiency enhanced survival and decreased peripheral parasitemia in murine malaria

**Fig 2:**
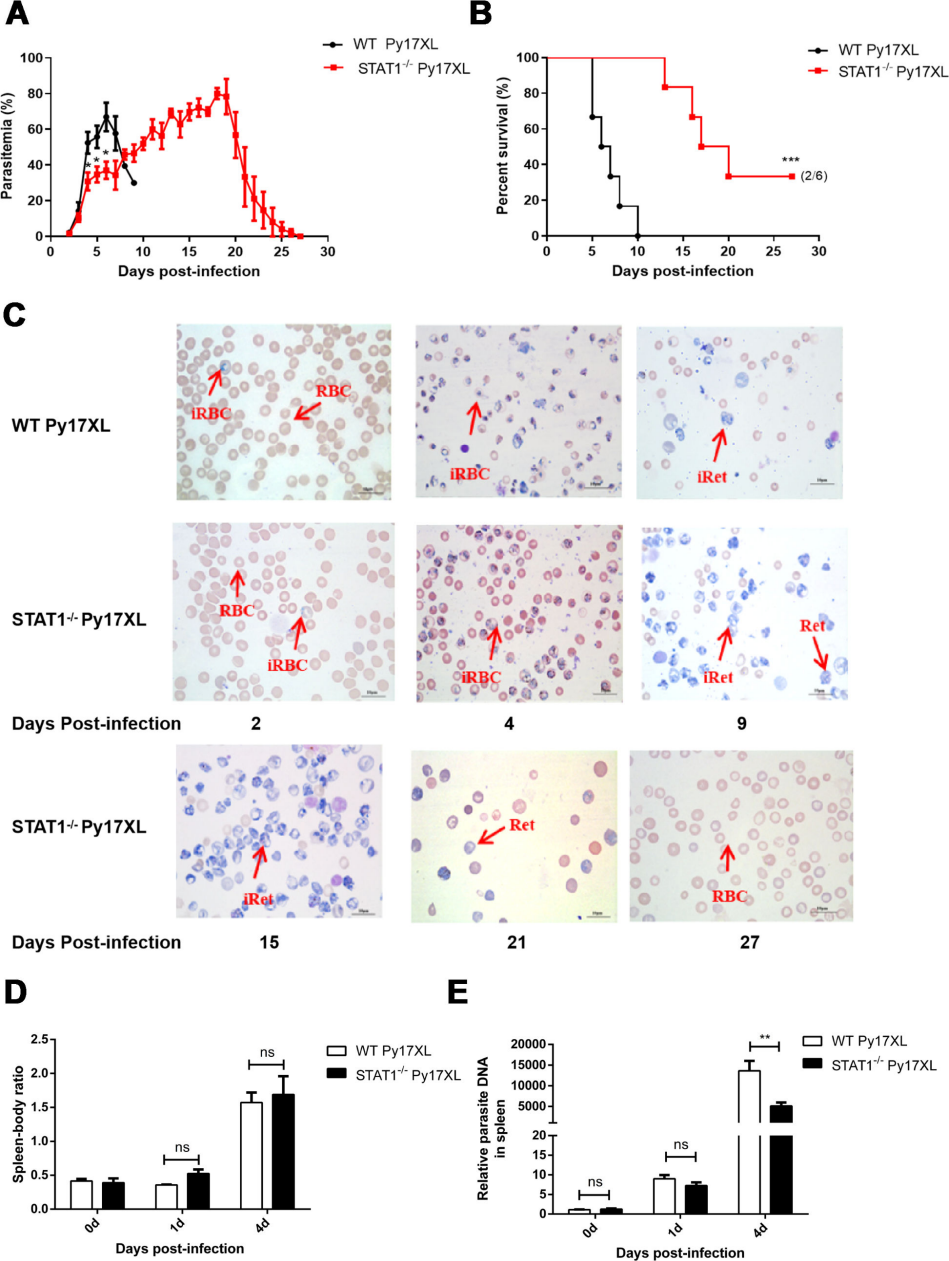
STAT1 deficiency enhanced mouse survival and decreased peripheral parasitemia in the murine malaria. WT and STAT1^−/−^ mice (*n* = 6/group) were infected with 1 × 10^6^ *Py*17XL iRBCs intraperitoneally. (**A**) Changes in the percentages of peripheral parasitemia in mice from days 0 to 27. (**B**) The survival rate of infected mice was monitored and recorded over 27 days. (**C**) Giemsa staining of peripheral blood smear from WT infected with *Py*17XL on 2, 4, and 9 days post-infection and STAT1^−/−^ mice on days 2, 4, 9, 15, 21, and 27 post-infection. Bar = 500 µm (**D**). Spleen body ratios were weighed and calculated on days 0, 1, and 4 post-infection. (**E**) Spleen parasite burden was determined by RT-qPCR on days 0, 1, and 4 post-infection; β-actin served as the loading control. The survival rates were represented with Kaplan–Meier curves, and the Mann–Whitney U-test was used to compare the differences between two groups of independent samples. **P < 0.01; ***P < 0.001; ns, no significance. Experiments were repeated 2–3 times.

To further elucidate the role of STAT1 in *Py*17XL-infected mice, a STAT1 KO murine malaria model was established. Notably, peripheral parasitemia was found to be significantly lower in STAT1 KO mice compared with that in wild-type (WT) mice at days 4, 5, and 6 post-infection (*P* = 0.0205, 0.0221, and 0.0123, respectively) (Fig. 2A). Furthermore, STAT1 KO mice exhibited prolonged survival up to 27 days, with a 33% survival rate (2/6 mice) during *Py*17XL infection (*P* = 0.0005) (Fig. 2B). In blood cell smears from *Py*17XL-infected STAT1 KO mice, remarkable increases in reticulocyte counts were observed, which possibly contributed to improved host survival (Fig. 2C). Although no differences were found in spleen-to-body weight ratios between WT and KO groups (Fig. 2D), splenic parasite burden was significantly lower in STAT1 KO mice than in WT mice at day 4 post-infection (*P* = 0.0099) (Fig. 2E). Moreover, in the spleens of STAT1-deficient mice, the STAT3 signaling was significantly activated, and pSTAT3 expression was gradually increased with the prolongation of *Py*17XL infection time (Fig. S1). Collectively, these findings indicate that STAT1 deficiency reduces peripheral parasitemia and enhances host survival, suggesting a pathogenic role for STAT1 in malaria infection.

### STAT1 deficiency regulated splenic IFN-γ and IL-10 production through T cells during malaria infection

**Fig 3:**
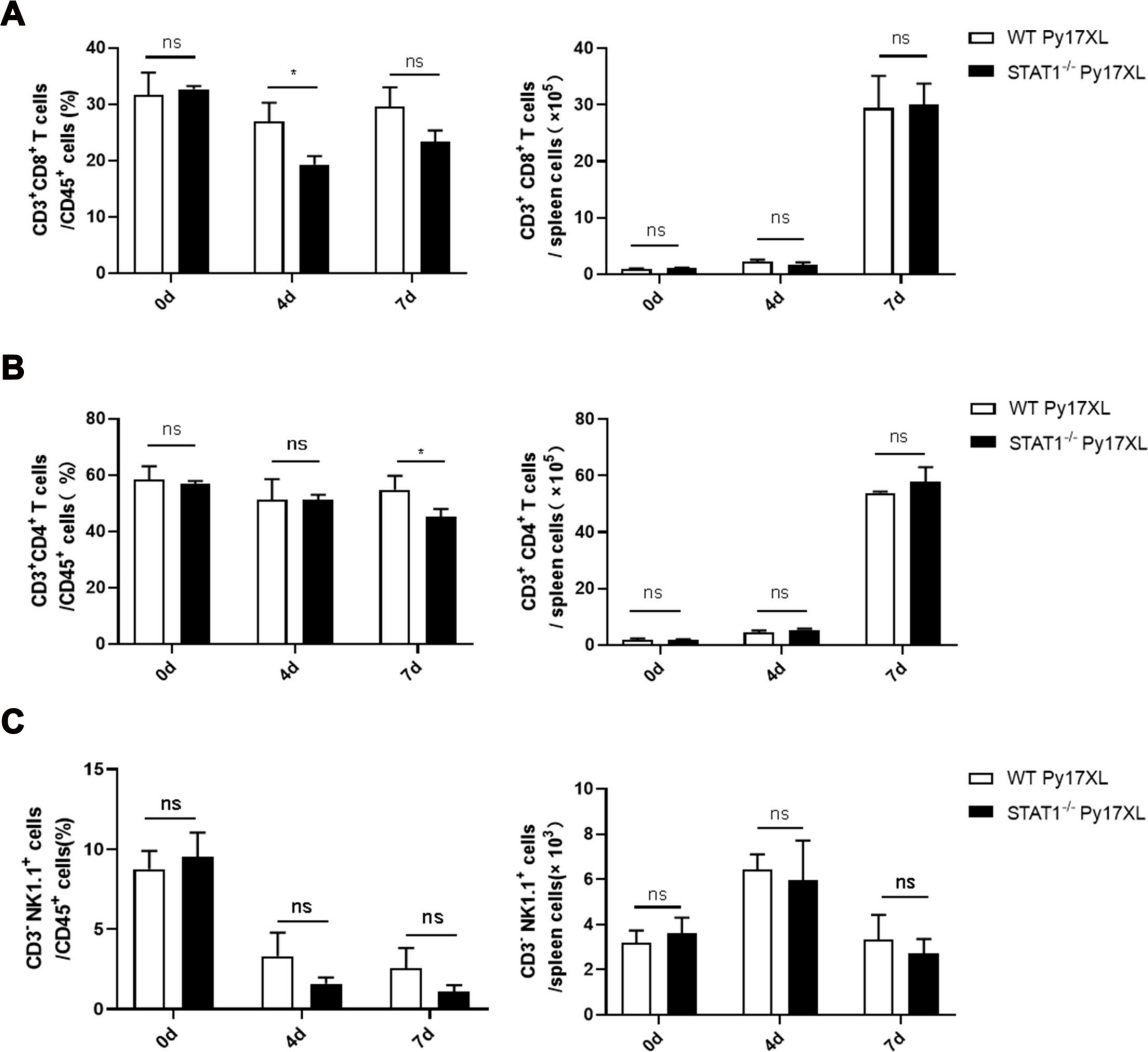
Downregulation of the proportion of T cells after STAT1 deletion in *Py-*infected mice (*n* = 6/group). (**A**) Proportion and absolute number of CD3^+^ CD8^+^ T cells (**A**), CD3^+^ CD4^+^ T cells (**B**), and CD3^-^ NK1.1^+^ cells (**C**) in the spleen of infected mice on days 0, 4, and 7 post-infection. The Mann–Whitney U-test was performed to compare the differences between two groups of independent samples. *P < 0.05; ns, no significance. Experiments were repeated 2–3 times.

To investigate the mechanisms underlying the pathogenic effects of STAT1, the impact of STAT1 deficiency was assessed in NK and T cell populations in host spleens. In *Py*17XL-infected mice, STAT1 KO significantly reduced proportions of CD8^+^ T cells at day4 post-infection (*P* = 0.0230) and CD4^+^ T cells at day 7 post-infection (*P* = 0.0426) (Fig. 3A and B; Fig. S2A), suggesting that STAT1 modulates T cell subset proportions during murine malaria. However, no significant differences were observed in NK cell abundance (*P* = 0.5192) or proportion (*P* = 0.1274) between STAT1 KO and WT mice at day 7 post-infection (Fig. 3C). These results suggest that STAT1 plays a crucial role in regulating T cell function.

**Fig 4:**
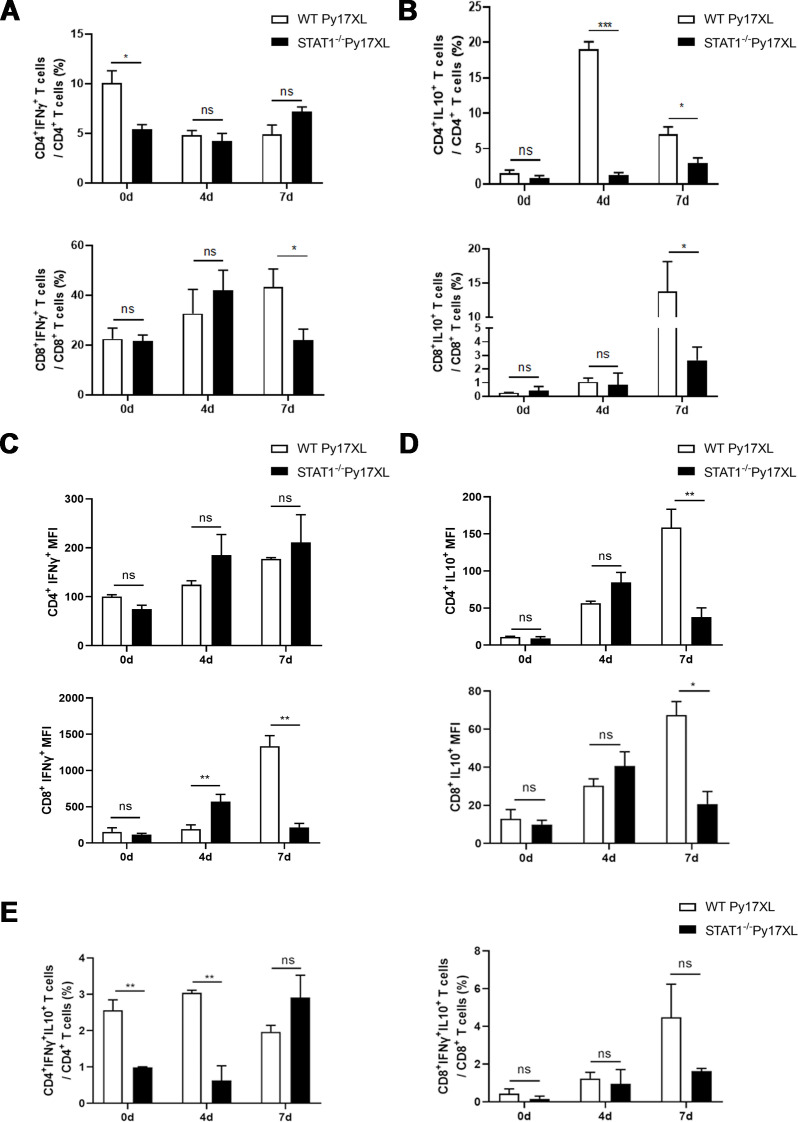
The deficiency of STAT1 affects the production of IFN-γ and IL-10 in T cells. (**A**) The proportions of CD3^+^ CD4^+^ IFN-γ^+^ T cells or CD3^+^ CD8^+^ IFN-γ^+^ T cells in the spleen from WT and STAT1^−/−^ mice (*n* = 6/group) infected with *Py*17XL. (**B**) The proportions of CD3^+^ CD4^+^ IL-10^+^ T cells or CD3^+^ CD8^+^ IL-10^+^ T cells in the spleen from WT and STAT1^−/−^ mice (*n* = 6/group) infected with *Py*17XL. (**C**) IFN-γ MFI from IFN-γ-producing CD4^+^ T cells or CD8^+^ T cells. (**D**) IL-10 MFI from IL-10-producing CD4^+^ T cells or CD8^+^ T cells. (**E**) The proportions of CD3^+^ CD4^+^ IFN-γ^+^ IL-10^+^ T cells or CD3^+^ CD8^+^ IFN-γ^+^ IL-10^+^ T cells in the spleen from WT and STAT1^−/−^ mice (*n* = 6/group) infected with *Py*17XL. The Mann–Whitney U-test was used to compare the differences between two groups of independent samples. *P < 0.05; **P < 0.01; ***P < 0.001; ns, no significance. Experiments were repeated 2–3 times.

To determine if STAT1 affects IFN-γ and IL-10 production via T cells, flow cytometry (FCS), along with gating strategies, was employed to analyze IFN-γ- and IL-10-producing T cells at days 0, 4, and 7 post-infection (Fig. S3). Although STAT1 deficiency significantly reduced the proportion of IFN-γ-producing CD4^+^ T cells in uninfected mice, no significant differences were observed at days 4 and 7 post-infection (Fig. 4A). Compared with WT mice, the proportion of IFN-γ-secreting CD8^+^ T cells in STAT1^−/−^ mice was significantly decreased at day 7 post-infection (Fig. 4A). In addition, the proportion of IL-10-secreting CD4^+^ T cells and CD8^+^ T cells was significantly lower in STAT1^−/−^ mice than in WT mice at day 7 post-infection (Fig. 4B). Further, the mean fluorescence intensity (MFI) of IFN-γ-producing CD8^+^ T cells in STAT1 KO mice was significantly lower than that in WT mice (Fig. 4C). While compared with control, lack of STAT1 significantly decreased the amounts of IL-10 (measured as MFI) produced by CD4^+^ T and CD8^+^ T cells in the spleen (Fig. 4D). Moreover, STAT1 KO exhibited no significant effect on IFN-γ-producing NK cells in the spleen at day 4 post-infection (Fig. S4). In the spleens of STAT1 KO mice, the proportion of IFN-γ and IL-10 coproducing-CD4^+^ T cells was significantly lower than that of WT mice at 0 and 4 days post-infection, while the proportion of IFN-γ and IL-10 coproducing-CD8^+^ T cells showed no significant difference between WT and STAT1 KO mice (Fig. 4E). Overall, these findings indicate that STAT1 promotes IFN-γ-producing CD8^+^ T cells and IL-10-producing CD4^+^ and CD8^+^ T cells, revealing its critical role in regulating IFN-γ and IL-10 production by T cells.

### STAT1 deficiency enhanced erythropoiesis in the blood stage of the murine malaria

**Fig 5:**
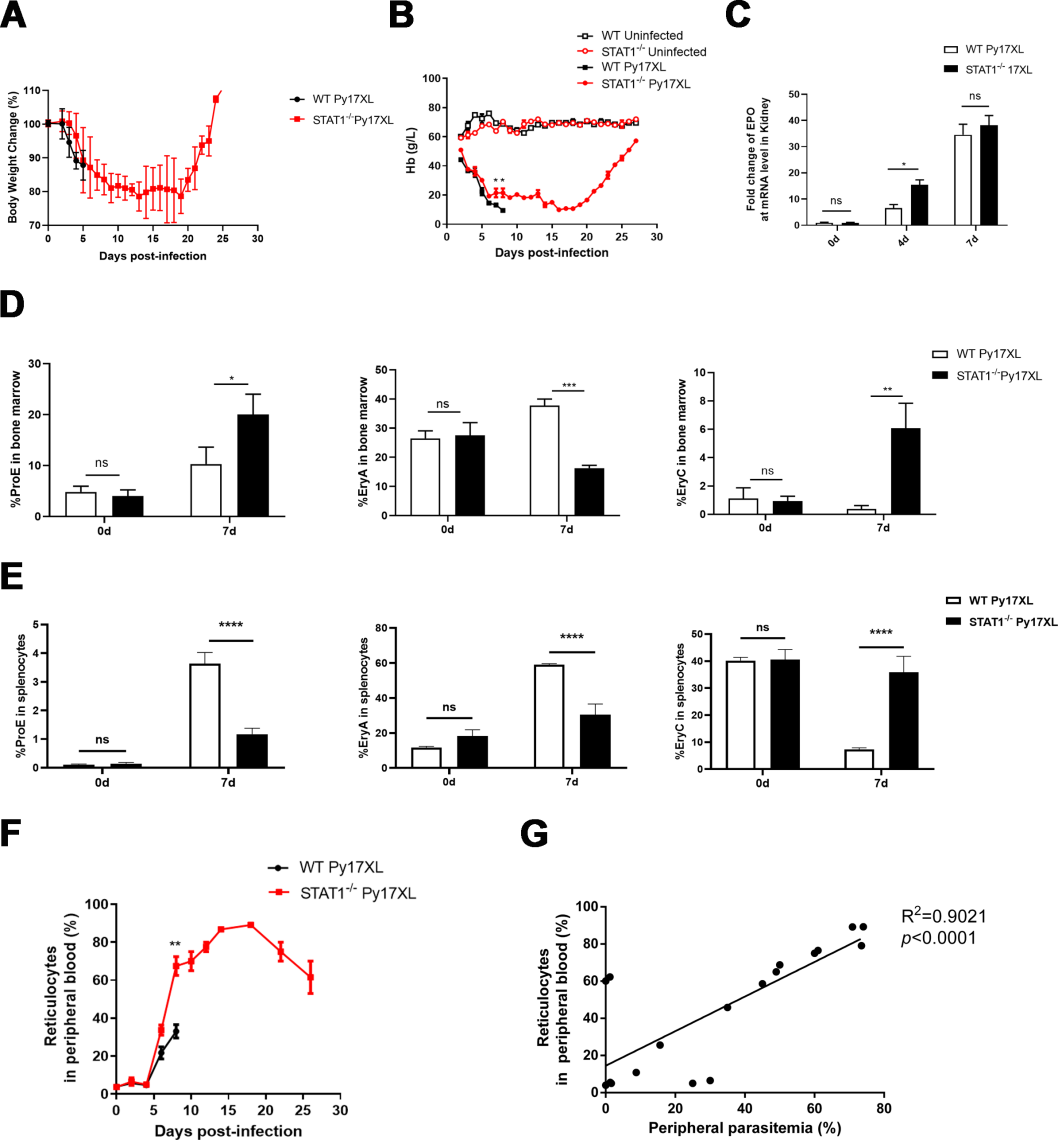
STAT1 deficiency enhanced the erythropoiesis in the blood stage of the murine malaria model. WT and STAT1^−/−^ mice (*n* = 6/group) were infected with 1 × 10^6^ *Py*17XL iRBCs intraperitoneally. (**A**) Changes in body weight from 0 to 27 days post-infection. (**B**) Hemoglobin concentration changes in the peripheral blood from 0 to 27 days post-infection. (**C**) The EPO mRNA levels in the kidneys of WT and STAT1^−/−^ mice infected with *Py*17XL were measured on days 0, 4, and 7 post-infection by RT-qPCR; β-actin served as the loading control. The proportion of PorE, EryA, and EryC in the bone marrow (**D**) and splenocytes (**E**) on days 0 and 7 post-infection in Py17XL infected-WT or STAT1^−/−^ mice. (**F**) The proportion of reticulocytes (Ter119^+^ CD71^+^ cells) in the peripheral blood until day 27 post-infection. (**G**) Correlation analysis between reticulocytes in the peripheral blood and peripheral parasitemia. The Mann–Whitney U-test was performed to compare the differences between two groups of independent samples. Correlation analysis was conducted using Pearson’s correlation test. *P < 0.05; **P < 0.01; ***P < 0.001; ****P < 0.0001; ns, no significance. Experiments were repeated 2–3 times.

STAT1 plays a pivotal role in erythropoiesis, and anemia is a common clinical manifestation of malaria (18). To explore the impact of STAT1 on malaria anemia, anemia-related parameters were evaluated in *Py*17XL-infected mice. Although no significant differences in body weight changes were observed between *Py*17XL-infected STAT1 KO and WT mice, body weight in surviving STAT1 KO mice gradually recovered up to day 20 post-infection (Fig. 5A). Compared with WT mice, Hb concentrations were significantly higher in STAT1 KO mice at days 7 (*P* = 0.0248) and 8 (*P* = 0.0471) post-infection, and the low peak of that was delayed. Hb levels in surviving STAT1 KO mice continued to rise after day 18 post-infection (Fig. 5B). Furthermore, STAT1 KO mice exhibited significantly elevated kidney erythropoietin (EPO) levels, an important indicator of erythropoiesis, at day four post-infection (*P* = 0.0223) (Fig. 5C). Ter119, the erythroid lineage marker, is induced in erythroblasts and persistently expressed during erythropoiesis, while CD71 (transferrin receptor 1) gradually decreases during the transition from reticulocytes to mature erythrocytes (19). Performing the CD71/Ter119 staining on bone marrow or spleen could identify four different subsets in the developmental sequence, which are labeled as ProE, EryA, EryB, and EryC (20). Compared with wild-type (WT) controls, STAT1 KO mice exhibited a significant increase in the proportion of proerythroblast (ProE) and most mature erythroblasts (EryC) in the bone marrow (Fig. 5D; Fig. S5). In stark contrast, within the spleen, the ProE population was significantly reduced in STAT1 KO mice, while the EryC proportion was again notably increased (Fig. 5E; Fig. S5). Additionally, STAT1^−/−^ mice showed significantly increased proportions of peripheral blood reticulocytes at day eight post-infection compared with those in WT mice (*P* = 0.0046) (Fig. 5F; Fig. S5), further confirming the erythropoiesis-promoting effect of STAT1 deficiency. To determine if *Plasmodium* parasites preferentially invade reticulocytes in STAT1 KO mice, the correlation between peripheral parasitemia and reticulocyte percentages was evaluated in the peripheral blood. Notably, peripheral parasitemia positively correlated with reticulocyte counts in *Py*17XL-infected STAT1 KO mice (*P* < 0.0001) (Fig. 5G). Collectively, these results demonstrate that STAT1 deletion enhances erythropoiesis in the bone marrow during murine malaria, indicating that STAT1 exacerbates malarial anemia.

## DISCUSSION

The spleen, as a pivotal immune organ during malaria infection, plays a crucial role in host–*Plasmodium* interactions and activates robust immune responses. Early transcriptomic studies revealed significant induction of IFN-related genes in the spleens of malaria-infected hosts. However, the functional enrichment of differentially expressed proteins in the spleen during *Plasmodium* infection required further proteomic analysis. Herein, upregulated proteins in the spleens of *Plasmodium*-infected hosts were found to be significantly enriched in IFN signaling pathways. Among them, STAT1 showed the largest number of unique peptides and served as the core molecule in protein–protein interaction networks. Notably, STAT1 deficiency improved survival rates, delayed peak peripheral parasitemia, and reduced splenic parasite burden in murine malaria models. Moreover, STAT1 KO significantly suppressed IFN-γ-producing CD8^+^ and IL-10-producing CD4^+^ T cells in the spleen. STAT1 deficiency also increased peripheral blood reticulocyte proportion, thereby alleviating malarial anemia. Overall, STAT1 promoted IFN-γ production and impaired erythropoiesis, contributing to malaria pathogenesis. Thus, the findings of this study elucidate key molecular mechanisms underlying the immunopathological role of spleen in malaria and identify novel intervention targets for anti-malarial drug development.

In this study, proteins involved in the IFN signaling pathway were significantly enriched, with STAT1 highly expressed in the spleens of mice infected with lethal *Py*17XL (Fig. 1). Previous single-cell transcriptomics study and clinical data have demonstrated elevated STAT1 expression in CD16^+^ monocytes within peripheral blood mononuclear cells from *Plasmodium falciparum*-infected patients (21). Consistently, multiple experimental malaria studies have confirmed significant increases in STAT1 transcriptional levels in both the spleen and liver (8, 9, 22). Moreover, elevated splenic STAT1 levels have been observed during both lethal and non-lethal strain infections using immunoblotting and immunohistochemistry in murine malaria models (7), consistent with the findings of the present study. Since the balance between pSTAT1 and pSTAT3 plays an essential role in maintaining homeostasis of host immune responses, a previous study had revealed the dynamic balance of pSTAT1 and pSTAT3 in *Plasmodium yoelii*-infected mice (23). Similarly, compared with uninfected controls, *Py*17XL-infected mice have shown a lower pSTAT1/pSTAT3 ratio, suggesting that lethal *Plasmodium* strains may inhibit T-helper 1 cell responses, leading to immunosuppression and ultimately death (7). Reportedly, STAT3 signaling regulates T follicular helper (Tfh) cell differentiation (24) and *Plasmodium*-induced Tfh cells promote anti-parasitic antibody production by B cells in both human and murine malaria models (25–27). Consequently, we speculate that STAT1 deficiency may enhance STAT3 activity, promoting Tfh cell differentiation and host protection (25). Further investigation is needed to identify specific cell types expressing STAT1 in Py-infected mice spleens. Additionally, significantly downregulated proteins identified through the proteomics screening may contribute to malaria pathogenesis. Future studies need to verify expression changes in these key downregulated proteins, determine their roles in malaria infection, and elucidate underlying mechanisms.

Importantly, the regulatory role of STAT1 in Py infection *in vivo* was investigated using a KO strategy. Multiple studies have employed STAT1 KO approaches to examine pathogen infection effects on host survival, immune responses, and pathological changes (6). STAT1 deficiency typically enhances susceptibility to viruses, increases viral loads, and causes notable pathological changes in tissues, including the liver, lung, and spleen (28–30). However, the present findings demonstrate a pathogenic role of STAT1 in malaria, contrasting its protective role in viral infections. Compared with WT mice, STAT1 KO mice exhibited significantly reduced peripheral parasitemia and prolonged survival in murine malaria models (Fig. 2A and B). It is worth noting that there is a large, hidden biomass of asexual-stage parasites accumulating in the spleen of human malaria patients (31). The results revealed that STAT1 KO reduced splenic parasite burden; however, because splenic parasite burden was measured using nucleic acid-based methods, this may also reflect dead parasite material remaining in the spleen. Interestingly, clinical data from Mali (a country in West Africa) showed that STAT1 expression was upregulated in peripheral blood mononuclear cells from children susceptible to clinical malaria compared to protected children before the transmission season, suggesting that pre-existing STAT1 expression differences influence malaria susceptibility (10). These findings corroborate the results of the present study, confirming the pathogenic effect of STAT1 in malaria infection.

Additionally, we further explored how STAT1 exacerbates pathogenesis in lethal Py-infected murine malaria models. IFN-γ, primarily produced by T cells and NK cells, plays a dual role in host immunity against malaria (11). The present results showed that STAT1 deficiency significantly reduced the proportion of IFN-γ-producing CD8^+^ T cells (Fig. 4C) but did not affect NK cells (Fig. S3). This led to the speculation that STAT1 may increase IFN-γ production to inhibit differentiation of Tfh and the B cell responses in germinal centers, ultimately resulting in insufficient antibody-secreting plasma cell production and weakened antibody responses needed to control malaria parasites. Interestingly, STAT1 KO also suppressed IL-10 production by CD8^+^ T cells at day 7 post-*Py*17XL infection (Fig. 4D). However, at 4 days post-infection, the amount of IFN-γ (MFI) produced by CD8^+^ T cells in STAT1-deficient mice was indeed significantly higher than that in the WT control (Fig. 4C). We speculate that due to the absence of STAT1, the signal cannot be transmitted to activate downstream pathways after the binding of IFN and its receptor within a short period of time. This results in a temporary increase in IFN production. To further explore the mechanism, future studies could employ cell depletion and antibody blocking strategy to determine whether the pathogenic role of STAT1 on malaria depends on IFN-γ producing-CD8^+^ T cells.

SMA, a life-threatening complication of malaria, represents a significant clinical manifestation of the disease (32). Presently, no effective treatments exist for SMA, highlighting the urgent need for novel intervention strategies based on underlying anemia mechanisms. STAT6, another STAT family member, has been shown to suppress erythropoiesis in experimental malarial anemia models (33). Our findings demonstrated that STAT1 KO increased the proportion of proerythroblast (ProE) and most mature erythroblasts (EryC) in bone marrow (Fig. 5D), suggesting that STAT1 may impair erythropoiesis to exacerbate malarial anemia. The spleen is considered a primary site for extramedullary hematopoiesis and part of the stress erythropoiesis. To further explore the impact of STAT1 on extramedullary erythropoiesis, CD71/Ter119 staining was used to study erythroid progenitors of spleen during *Plasmodium* infection. Our results showed that STAT1 deficiency reduces ProE proportion while markedly increasing EryC proportion in the spleen (Fig. 5E). Notably, ablation of acid ceramidase (Ac) also led to dysregulated erythropoiesis and alleviated parasitemia in *Plasmodium yoelii* infection (34). TLR7 deficiency exacerbates malarial anemia and impairs stress erythropoiesis in the spleen (35). TLR7 promotes extramedullary erythropoiesis primarily through the modulation of iron metabolism, thereby supporting erythroid progenitor expansion in the spleen during infection (35). In contrast, STAT1 may function as a regulator to extramedullary early erythropoiesis, thereby influencing the stress-induced erythropoiesis during infection.

Importantly, patients with GOF-STAT1 mutations frequently suffer from severe anemia, associating STAT1 overexpression with anemia (15, 17, 36). For instance, an etiological study found that STAT1 overexpression leads to bone marrow failure and aplastic anemia (17). Moreover, STAT1 levels positively correlate with anemia in SLE patients (16). Although there are many factors contributing to clinical anemia, dyserythropoiesis is an important one among them. Early studies have critically implicated STAT1 in regulating erythropoiesis. For example, in STAT1 KO mice, splenic burst-forming units and colony-forming units in the bone marrow have been shown to exhibit compensatory increases (37). These findings align with the present observations of improved proportions of PrE and EryC cells in STAT1 KO mice. Furthermore, as inflammatory anemia constitutes a key mechanism of malaria anemia (38), it is necessary to determine that the inflammatory response triggered by STAT1 exacerbates the malarial anemia. In fact, IL-10 plays a dual role in malaria-induced anemia. IL-10 counteracts the pathological effects of TNF, protecting against inflammation-driven anemia. IL-10 may also suppress Th1-mediated immunity, impairing parasite clearance and indirectly exacerbating anemia (39). Our study reveals that STAT1 upregulates IL-10-producing CD4^+^ and CD8^+^ T cells. Therefore, STAT1 may exacerbate malaria anemia by upregulating IL-10 expression. Whether STAT1 affects malaria-induced anemia by regulating IL-10 remains to be determined in the future.

In conclusion, this study identifies STAT1 as a key upregulated protein in malaria-infected spleens through proteomic screening and elucidates its pathogenic effects via classic signaling molecule STAT1 in *Py*17XL infection, including exacerbation of parasitemia and anemia. STAT1 KO significantly improved mouse survival, reduced peripheral and splenic parasitemia, downregulated IFN-γ production by CD8^+^ T cells, and suppressed IL-10 production by CD4^+^ T cells during Py infection. Additionally, STAT1 deficiency ameliorated malaria anemia by regulating erythropoiesis. Overall, these findings deepen our understanding of dynamic protein changes in the host spleen during malaria and provide a research foundation for developing novel malarial anemia therapies.

## MATERIALS AND METHODS

### Mice and parasites

Female BALB/c and C57BL/6J mice were purchased from GemPharmatech Co., Ltd. (Nanjing, Jiangsu, China). Since BALB/c mice are highly susceptible to *Py* 17XL, exhibiting rapid parasitemia progression and pronounced pathology (40), they were used for the proteomic analysis. STAT1 KO mice (STAT1^−/−^) (C57BL/6J background [41]) were generously provided by Professor Zhou Rongbin (University of Science and Technology of China, Hefei, China). All mice were housed under specific pathogen-free conditions at the Laboratory Animal Center of the Jiangsu Institute of Parasitic Diseases in a suitable environment with a 12-h light/dark cycle at 22°C ± 3°C.

### Animal genotyping and PCR

Mouse genotyping was performed as described previously (42) using genomic DNA extracted using the Universal Genomic DNA Kit (CWBIO, China). PCR amplification was conducted using the 2×Taq Plus Master Mix (CWBIO) under the following conditions: initial denaturation at 98°C for 3 min; 35 cycles of 98°C for 10 s, 55°C for 30 s, and 72°C for 30 s; followed by final extension at 72°C for 5 min. WT mice were identified with the primers: 5′-CTACCAGAGTATCTGCCTAGAC-3′ (sense) and 5′-CCTCTCAACCTTCCTGACACC-3′ (antisense). STAT1^−/−^ mice were identified with primers: 5′-CTACCAGAGTATCTGCCTAGAC-3′ (sense) and 5′-CGCCGCTCCCGATTCGCAGCGCATCGC-3′ (antisense).

### Establishment of a murine malaria model

To establish a murine malaria model of infection with *Py*17XL, cryopreserved blood from *Py*17XL-infected mice was passaged via intraperitoneal injection into mice at 6–8 weeks of age. After the mice were infected with *Py*17XL, their tail blood smears were collected to prepare thin blood smears and stained using the Giemsa Stain Kit (Jiancheng, China). Five fields (approximately 1,000 infected RBCs) were randomly selected and observed under a microscope. Then, each of the thin blood smear specimens was photographed, and the proportions of infected RBCs in the total RBCs were enumerated. The parasitemia of the mice was monitored every day until death or at the time of sacrifice. When peripheral parasitemia reached approximately 60%, blood was collected into sterile PBS and prepared for inoculating into the experimental mice. Female mice were intraperitoneally injected with 1 × 10⁶ *Py*17XL parasite-infected red blood cells. Uninfected mice served as controls. To investigate the role of STAT1 in malaria, C57BL/6J WT and STAT1 KO mice were used to establish a murine malaria model. The spleens of the animals were removed after sacrificing them in accordance with the experimental ethics requirements, and the spleen-to-body ratio was calculated as spleen weight (grams) per gram of body weight. The body weight changes of the mice were monitored daily.

### Proteomics and bioinformatics

Spleen tissue samples from uninfected and *Py*17XL-infected BALB/c mice were subjected to cryogenic grinding using liquid nitrogen. The resulting powder was transferred into a 50-mL centrifuge tube and stored at −80°C. Subsequent proteomic analysis was conducted by Jingjie PTM Biolabs. The samples were digested with trypsin (1:50 trypsin-to-protein mass ratio for the initial overnight digestion and 1:100 trypsin-to-protein mass ratio for the subsequent 4-h digestion). The resulting peptides were analyzed by liquid chromatography-tandem mass spectrometry (LC-MS/MS). Bioinformatics analysis (performed by Jingjie PTM Biolabs) included functional annotation using Gene Ontology (GO) enrichment analysis and Kyoto Encyclopedia of Genes and Genomes (KEGG) pathway analysis. In addition, enrichment-based clustering and protein–protein interaction analysis was conducted to further elucidate protein function and relationships.

### Western blotting

The spleen tissues (20 mg) were lysed on an ice bath with 200 µL of RIPA lysis buffer (Beyotime Biotech, China) containing phenylmethylsulfonyl fluoride (PMSF) and phosphatase inhibitors for 30 min. The samples were vortexed every 10 min and centrifuged at 12,000 × *g* for 15 min at 4°C. The supernatant was collected, and the protein concentration was determined by using a BCA assay kit (Meilun Biotech, Dalian, China). Proteins (20 μg) were separated by 10% sodium dodecyl sulfate-polyacrylamide gel electrophoresis (SDS-PAGE) and transferred onto a polyvinylidene difluoride (PVDF) membrane (Immobilon, Millipore, USA). The membrane was blocked with 5% skim milk and washed with Tris-buffered saline containing 0.1% Tween-20 (TBS-T). Primary antibodies against STAT1, p-STAT1, STAT3, and pSTAT3 (Cell Signaling Technology, USA) were incubated with the membrane at a 1:1,000 dilution overnight at 4°C. A horseradish peroxidase (HRP)-conjugated goat anti-rabbit IgG secondary antibody (Southern Biotech, USA) was used for detection at a 1:5,000 dilution for 1 h at room temperature (RT). Chemiluminescent detection was performed using the NcmECL Ultra kit (NCM Biotech, China) and imaged with a ChemiDoc MP imaging system (Bio-Rad, USA). The experiments were conducted in triplicate, and the blot intensities were quantified using ImageJ (NIH, USA).

### Assessment of the spleen parasite burden

The spleen parasite burden was assessed by real-time quantitative polymerase chain reaction (RT-qPCR) targeting parasite 18S ribosomal RNA (rRNA) in mice infected with *Py*17XL. Total RNA was extracted from the spleen at specified time points post-infection by using the RNA Quick Purification kit (Yishan Biotech, China). The extracted RNA was then subjected to reverse transcription by using the PrimeScript RT reagent Kit (TAKARA, Japan). For RT-qPCR, SYBR Premix Ex Taq II (TAKARA) was used as per the manufacturer’s instructions for *Plasmodium* 18S rRNA. The reaction was monitored using the LightCycle 480 II system (Roche, Switzerland). Data were normalized to β-actin control for each sample and presented as 2^−ΔΔCt^ values. Primers used for RT-qPCR are listed in Table S1.

### RNA extraction and real-time quantitative PCR

RNA extraction and RT-qPCR were performed as described in the “Assessment of spleen parasite burden” section. The primers for murine erythropoietin (EPO) are provided in Table S1.

### FCS analysis of splenic immune cells and cytokines

After the WT and STAT1^−/−^ mice were killed, the spleen was removed, mechanically ground, and filtered with a 70-μm filter (Solarbio, China). The red blood cells were lysed with RBC lysis buffer (Beyotime, China), and the splenocytes were resuspended in RPMI 1640 (Meilun Biotech). FC receptors were blocked with anti-mouse CD16/32 (BioLegend, USA) before staining. After washing with phosphate-buffered saline (PBS), the cells were stained with antigen-specific coupling antibodies for 30 min in the dark at 4°C. These antigens included Percp/Cy5.5-CD45 (BioGems, USA), PE-cy7-CD3 (BioGems), BG-Violet 500-CD4 (BioGems), FITC-anti-CD8 (BioLegend), and PE-anti-NK1.1 (BioLegend). The stained cells were analyzed by FCS (BD FACSAria III), and the data were analyzed using FlowJo software. For intracellular cytokine staining, PMA/Ionomycin Mixture (Yishan) and BFA/Monensin Mixture (Yishan) were added to the single cell suspension for 0.5 h at 37°C in a 5% CO_2_ incubator. Following incubation, the cells were collected and washed twice with PBS. Viability staining was performed using Live/Dead Fixable Dyes (BioLegend) for 20 min in the dark. The cells were washed with PBS, stained with antigen-specific coupling antibody on the cell surface for 30 min in the dark at 4°C, and then re-washed with PBS. Next, the cells were fixed with a fixation solution for 10 min at RT in the dark, permeabilized with 0.1% Triton X-100 for 10 min, and then washed three times with PBS, followed by staining with cytokine-specific coupling antibody for 30 min in the dark at 4°C. Isotype-matched cytokine and fluorescence minus one (FMO) control were included in each staining protocol. These antigens included APC-anti-IFN-γ and PE-anti-IL-10 (BioLegend). The stained cells were analyzed by FCS (BD FACSAria III), and the data were analyzed using FlowJo software.

### Hemoglobin and hematologic blood parameters detection

The hemoglobin levels in the uninfected and infected mice were quantified by using a hemoglobin detection kit (Jiancheng, China) according to the manufacturer’s instructions. Briefly, 2 µL of mouse tail blood was mixed with the detection reagent for 5 min, and the absorbance was measured at 540 nm by using a Multi-Detection Microplate reader (BioTek, USA).

### Detection of reticulocytes in the peripheral blood and bone marrow by FCS

To detect reticulocytes in the peripheral blood, tail blood was collected in an Eppendorf (EP) tube (1.5 mL) containing PBS and heparin. The cells were stained with FITC-Ter119 and PE-CD71 (BioLegend) for 30 min at 4°C, re-washed, and resuspended in PBS. FCS was conducted using the BD FACSAria III. Data were analyzed using FlowJo software.

For reticulocyte detection in the bone marrow, the bone marrow cells were flushed from the tibias and femurs with RPMI 1640 incomplete medium. The staining protocol for FCS PE-CD71 and FITC-Ter119 (BioLegend) was kept the same as that for peripheral blood red blood cells. The cells were fixed with a fixation buffer for 10 min at RT and washed three times with PBS. Isotype-matched cytokine and FMO control were included in each staining protocol. The samples were analyzed on the BD FACSAria III FCS. The data were analyzed using the FlowJo software.

WT and STAT1^−/−^ mice from uninfected controls and *Py*17XL-infected groups at days 4 and 7 post-infection were euthanized, and spleens were harvested. Single-cell suspensions were prepared by mechanical dissociation through a 40-μm cell strainer, followed by centrifugation at 300 × *g* for 5 min at RT. Cells were adjusted to 1 × 10⁶ cells/mL, washed twice with PBS, and incubated with anti-mouse CD16/32 antibody (2 μL per tube) for 10 min at 4°C to block Fc receptors. Cells were then stained with PE-anti-mouse CD71 and FITC-anti-mouse Ter119 antibodies for 30 min at 4°C in the dark. After washing, cells were analyzed on a BD Accuri C6 flow cytometer, and data were processed using FlowJo software.

### Statistical analysis

Statistical analyses and data visualization were performed using GraphPad Prism software (version 5.0). The gray values of protein bands were quantified using ImageJ (National Institutes of Health, USA). A log-rank test was performed for the analysis of mouse survival, and the survival rates were represented with Kaplan–Meier curves. The nonparametric Mann–Whitney *U*-test was applied to compare the two groups. Multiple group comparisons were assessed using ANOVA with appropriate post-hoc tests. Correlation analysis was conducted using Pearson’s correlation test. All statistical tests were two-tailed, with *P* < 0.05 considered to indicate statistical significance.

## SUPPLEMENTAL MATERIAL

Figure S1 spectrum.03032-25-s0001.tif Expression of STAT3 and pSTAT3 was detected by western blotting in WT and STAT1^−/−^ mice infected with *Py*17XL on days 0, 1, and 4 post-infection.

Figure S2 spectrum.03032-25-s0002.tif Representative gating strategies of T cells and NK cells in WT and STAT1^−/−^ mice infected with *Py*17XL.

Figure S3 spectrum.03032-25-s0003.tif Representative gating strategies for the detection of T cell-produced IFN-γ and IL-10 in WT and STAT1^−/−^ mice infected with *Py*17XL.

Figure S4 spectrum.03032-25-s0004.tif IFN-γ produced by NK cells in WT and STAT1^−/−^ mice infected with *Py*17XL on days 0, 1, and 4 post-infection.

Figure S5 spectrum.03032-25-s0005.tif Representative gating strategies for erythropoiesis in the peripheral blood (A), bone marrow (B), and spleen (C).

Supplemental legends spectrum.03032-25-s0006.docx Legends for Figures S1 to S5.

Table S1 spectrum.03032-25-s0007.docx Primer sequence of RT-qPCR for Py18S and murine EPO.

ASM does not own the copyrights to Supplemental Material that may be linked to, or accessed through, an article. The authors have granted ASM a non-exclusive, world-wide license to publish the Supplemental Material files. Please contact the corresponding author directly for reuse.
