# Supplementary figures and images for: Role of STAT1 in modulating the host immune response to Plasmodium yoelii 17XL-infected murine blood-stage malaria
Source: Microbiol Spectr. 2026 Mar 30;14(5):e03032-25. doi: 10.1128/spectrum.03032-25 (PMC13142038; doi:10.1128/spectrum.03032-25)

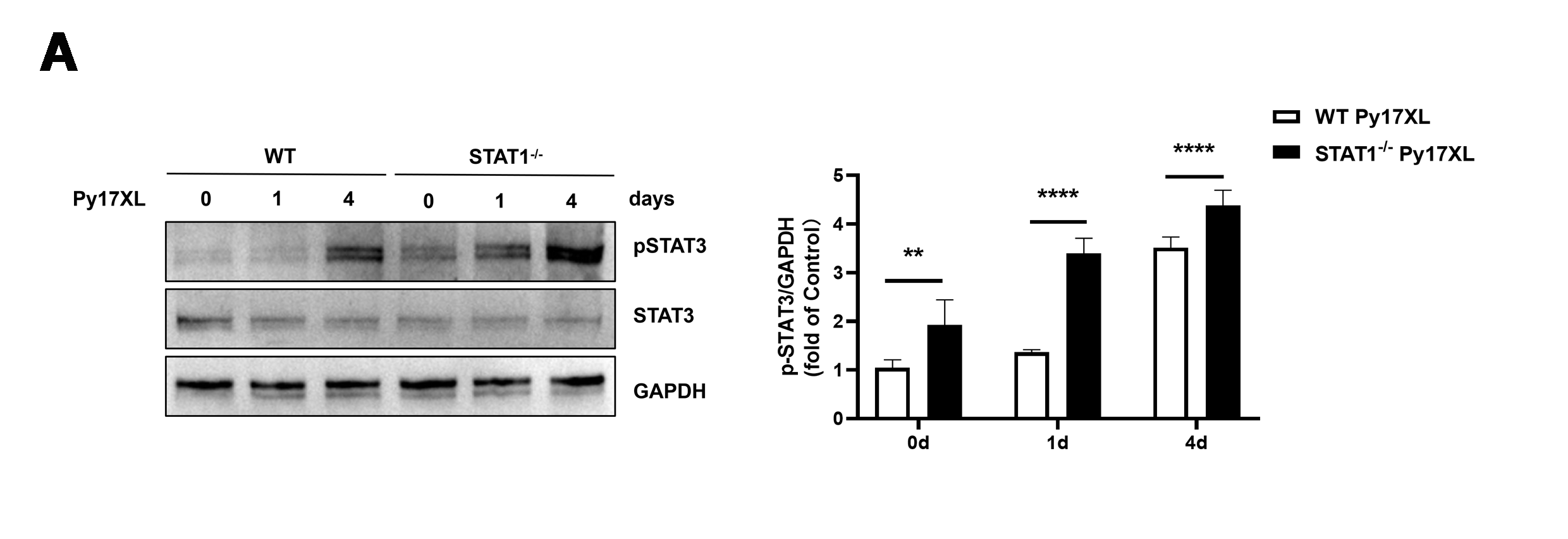

Supplement: Figure S1 — Expression of STAT3 and pSTAT3 was detected by western blotting in WT and STAT1−/− mice infected with Py17XL on days 0, 1, and 4 post-infection. [file spectrum.03032-25-s0001.tif]

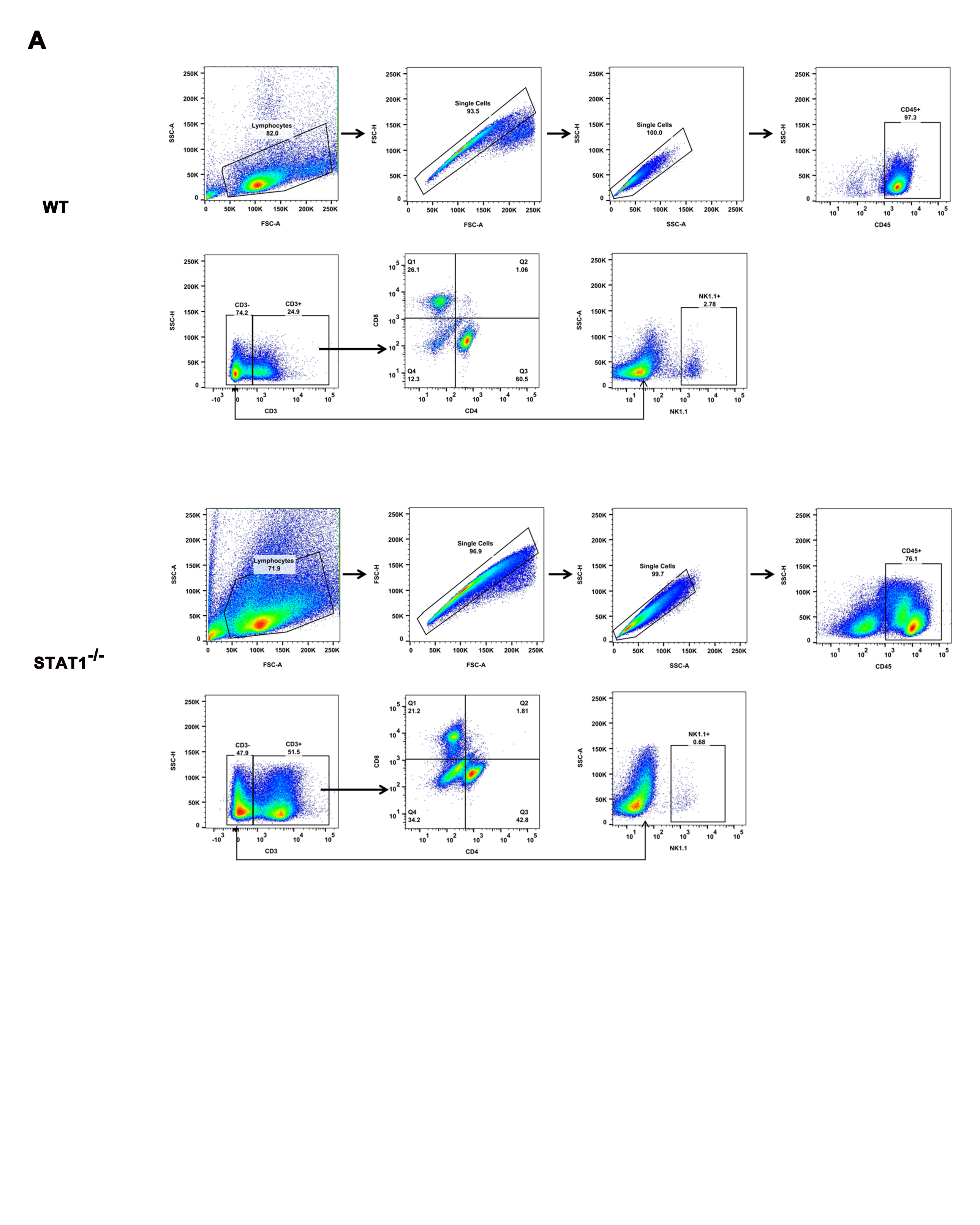

Supplement: Figure S2 — Representative gating strategies of T cells and NK cells in WT and STAT1−/− mice infected with Py17XL. [file spectrum.03032-25-s0002.tif]

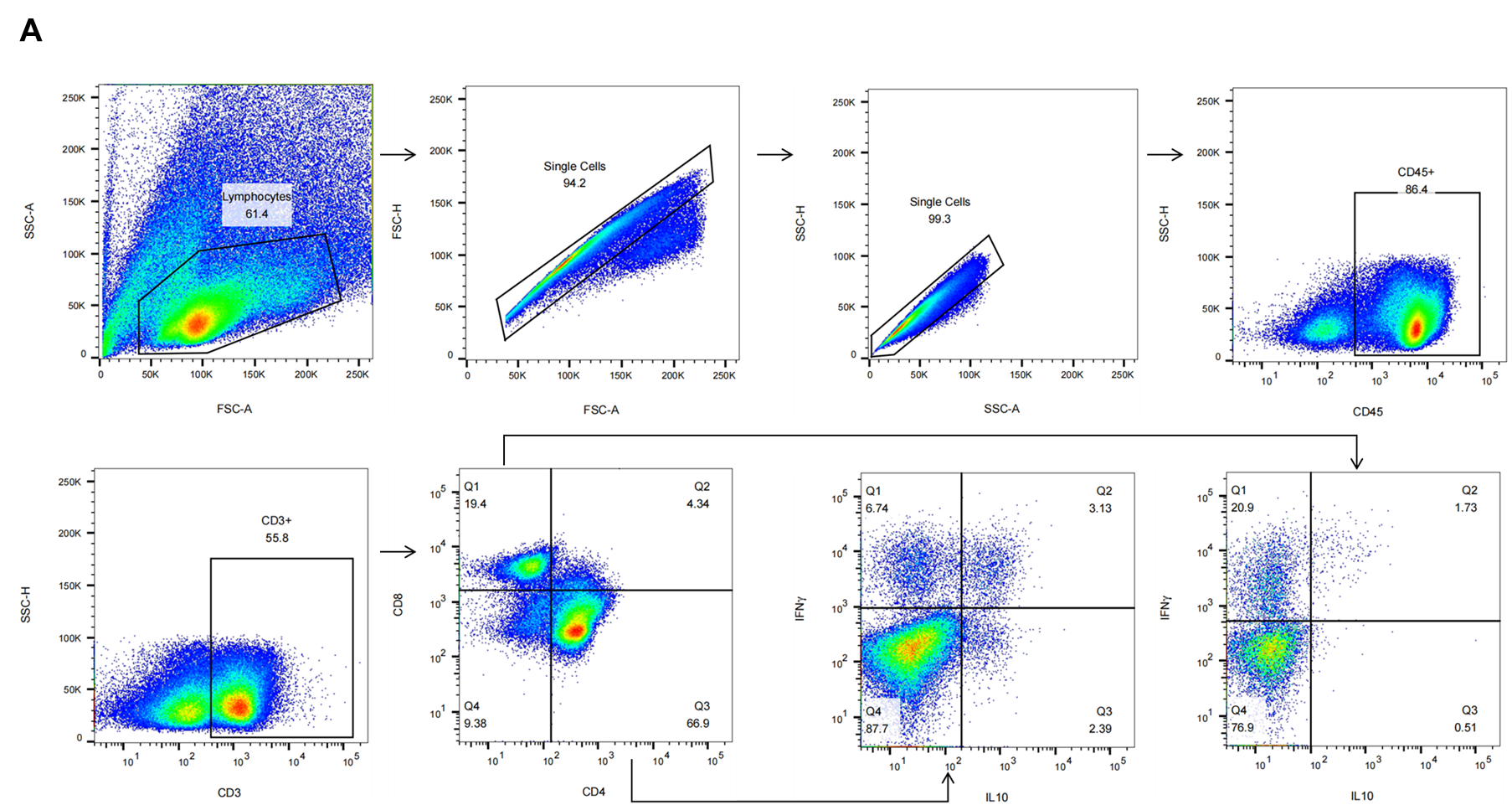

Supplement: Figure S3 — Representative gating strategies for the detection of T cell-produced IFN-γ and IL-10 in WT and STAT1−/− mice infected with Py17XL. [file spectrum.03032-25-s0003.tif]

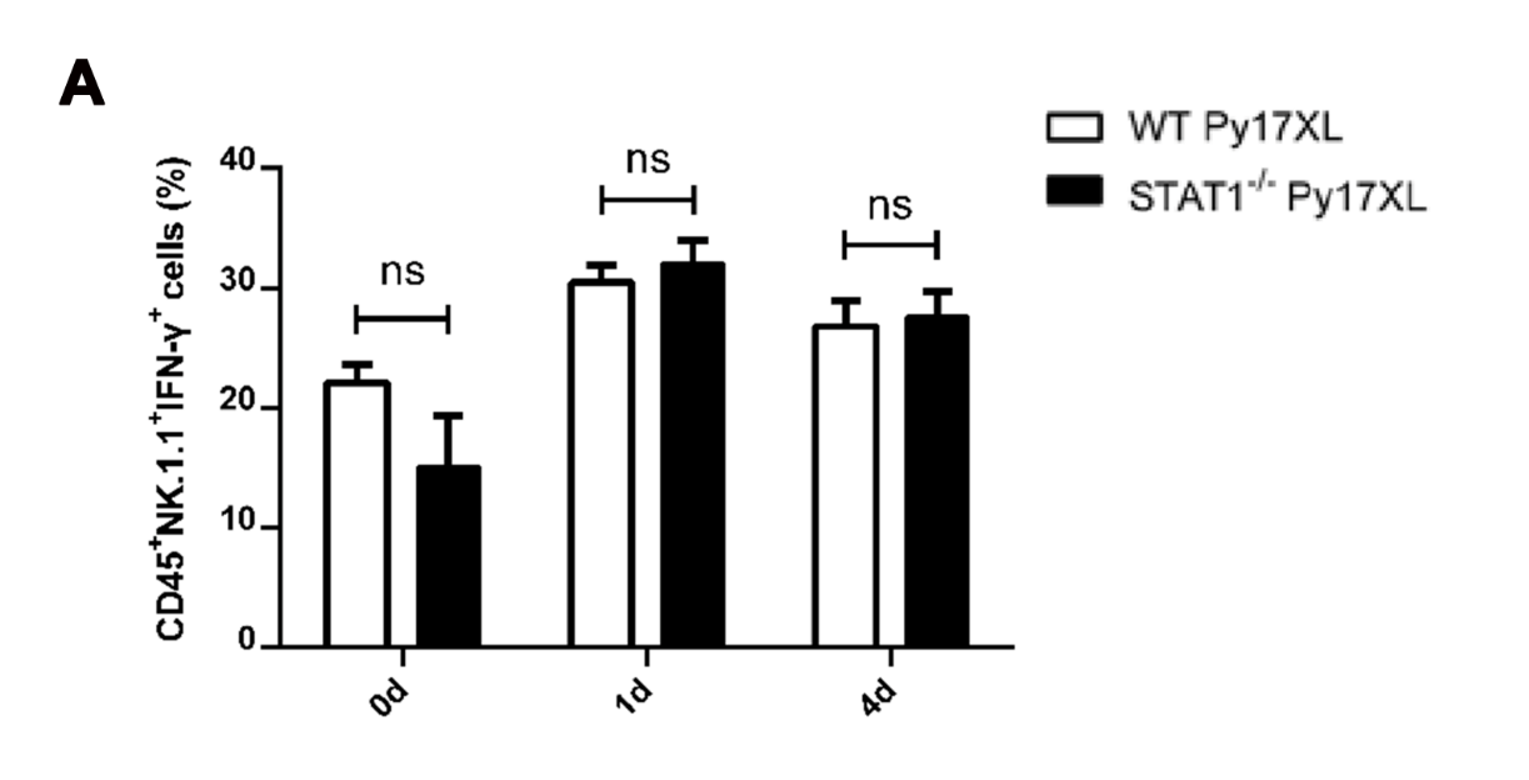

Supplement: Figure S4 — IFN-γ produced by NK cells in WT and STAT1−/− mice infected with Py17XL on days 0, 1, and 4 post-infection. [file spectrum.03032-25-s0004.tif]

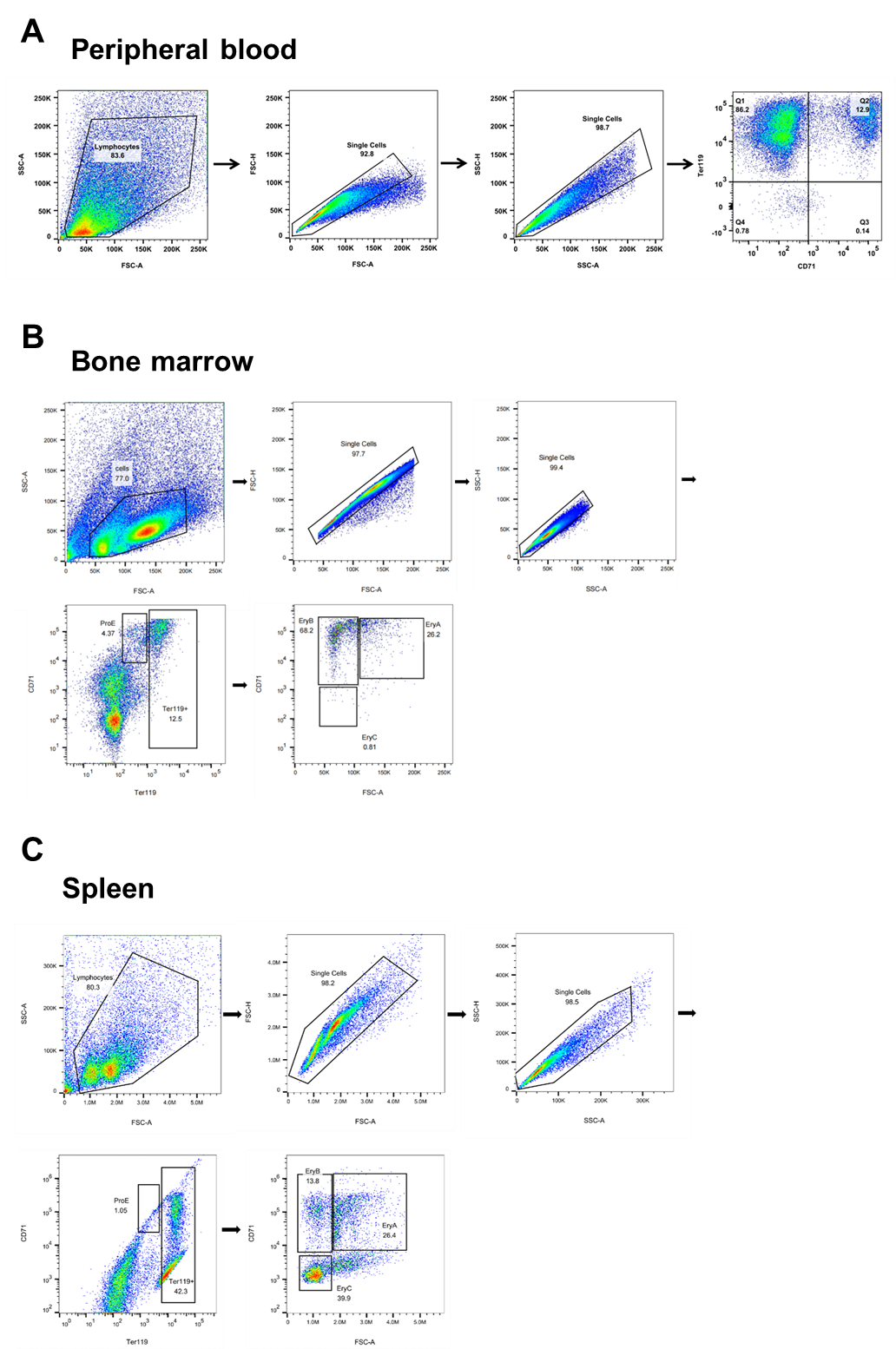

Supplement: Figure S5 — Representative gating strategies for erythropoiesis in the peripheral blood (A), bone marrow (B), and spleen (C). [file spectrum.03032-25-s0005.tif]
