# Supplementary material for: Role of STAT1 in modulating the host immune response to Plasmodium yoelii 17XL-infected murine blood-stage malaria
Source: Microbiol Spectr. 2026 Mar 30;14(5):e03032-25. doi: 10.1128/spectrum.03032-25 (PMC13142038; doi:10.1128/spectrum.03032-25)
Supplement: Supplemental legends — Legends for Figures S1 to S5. [file spectrum.03032-25-s0006.docx]

**Supplemental legends for Supplemental Figures**

**Fig. S1** Expression of STAT3 and pSTAT3 was detected by western blotting in WT and STAT1^−/−^ mice infected with Py17XL on days 0, 1, and 4 post-infection. Statistical analysis of the gray values of the pSTAT3 expression in total protein; GAPDH served as a quantitative control for analysis. Gray values of the protein bands were quantified using ImageJ. One-way ANOVA was used for statistical comparisons. ***p* < 0.001, *****p* < 0.0001. Experiments were repeated 3-4 times.

**Fig. S2** Representative gating strategies of T cells and NK cells in WT and STAT1^−/−^ mice infected with *Py*17XL. Dead cells, aggregates, and non-lymphoid cells were excluded from the study.

**Fig. S3** Representative gating strategies for the detection of T cell-produced IFN-γ and IL-10 in WT and STAT1^−/−^ mice infected with *Py*17XL. Dead cells, aggregates, and non-lymphoid cells were excluded from the study.

**Fig. S4** IFN-γ produced by NK cells in WT and STAT1^−/−^ mice infected with *Py*17XL on days 0, 1, and 4 post-infection. The Mann–Whitney U-test was performed to compare the differences between two groups of independent samples. ns, no significance.

**Fig. S5** Representative gating strategies for erythropoiesis in the peripheral blood (A), bone marrow (B), and spleen (C). Dead cells, aggregates, and non-lymphoid cells were excluded from the study.
