# Supplementary material for: Role of STAT1 in modulating the host immune response to Plasmodium yoelii 17XL-infected murine blood-stage malaria
Source: Microbiol Spectr. 2026 Mar 30;14(5):e03032-25. doi: 10.1128/spectrum.03032-25 (PMC13142038; doi:10.1128/spectrum.03032-25)
Supplement: Table S1 — Primer sequence of RT-qPCR for Py18S and murine EPO. [file spectrum.03032-25-s0007.docx]

**Supplementary Information**

**Table S1** Primer sequence of RT-qPCR for Py18S and murine EPO.

| **Primers** | **Sequences** |
| --- | --- |
| Py18S rRNA-Forward | GGGGATTGGTTTTGACGTTTT TGCG |
| Py18S rRNA-Reverse | AAGCATTAAATAAAGCGAATACATCCTTAT |
| murine β-actin-Forward | GGCTGTATTCCCCTCCATCG |
| murine β-actin-Reverse | CCAGTTGGTAACAATGCCATGT |
| murine EPO-Forward | CGCTTGGAAGACTTGGTGGTG |
| murine EPO-Reverse | CCTGGTGCAGGCTACATGAC |
